# Liver Derived FGF21 Maintains Core Body Temperature During Acute Cold Exposure

**DOI:** 10.1038/s41598-018-37198-y

**Published:** 2019-01-24

**Authors:** Magdalene Ameka, Kathleen R. Markan, Donald A. Morgan, Lucas D. BonDurant, Sharon O. Idiga, Meghan C. Naber, Zhiyong Zhu, Leonid V. Zingman, Justin L. Grobe, Kamal Rahmouni, Matthew J. Potthoff

**Affiliations:** 1 Department of Pharmacology, University of Iowa Carver College of Medicine, Iowa City, IA 52242 USA; 2 Fraternal Order of Eagles Diabetes Research Center, University of Iowa Carver College of Medicine, Iowa City, IA 52242 USA; 3 Department of Internal Medicine, University of Iowa Carver College of Medicine, Iowa City, IA 52242 USA; 4 Obesity Research and Education Initiative, University of Iowa Carver College of Medicine, Iowa City, IA 52242 USA; 5 Department of Veterans Affairs Medical Center, Iowa City, IA 52242 USA

## Abstract

Fibroblast Growth Factor 21 (FGF21) elicits an array of metabolic effects. However, the physiological role of FGF21 during thermal challenges is not clear. In this study, we assessed the tissue source of FGF21 and its site of action to regulate core body temperature in response to cold. Using mice lacking FGF21 specifically in the liver (FGF21 LivKO) or adipose tissues (FGF21 AdipoKO), we performed a series of cold exposure studies to examine the tissue specific induction of FGF21 in response to cold. We also examined the physiological site of FGF21 action during cold exposure by impairing FGF21 signaling to adipose tissues or the central nervous system (CNS) using genetic ablation of the FGF21 co-receptor β-klotho in adipose tissues (KLB AdipoKO) or pharmacological blockage of FGF21 signaling. We found that only liver-derived FGF21 enters circulation during acute cold exposure and is critical for thermoregulation. While FGF21 signaling directly to adipose tissues during cold is dispensable for thermoregulation, central FGF21 signaling is necessary for maximal sympathetic drive to brown adipose tissue to maintain thermoregulation during cold. These data demonstrate a previously unrecognized role for FGF21 in the maintenance of body temperature in response to cold.

## Introduction

Maintenance of core body temperature is a critical homeostatic factor regulating physiological processes and survival. Reductions in core body temperature can affect membrane fluidity, ion fluxes, and enzymatic reactions which may lead to significant consequences for an organism^1^. To prevent reductions in core body temperature in response to thermal challenges (i.e., cold), fundamental neural circuits are activated by thermal receptors which sense changes in either the ambient or internal environment. These thermoregulatory pathways then orchestrate behavioral and autonomic responses that produce alterations in core body temperature^2,3^. In many mammals, thermogenesis, or the production of heat, by brown adipose tissue (BAT) is a critical component of the homeostatic machinery to maintain body temperature^3–5^. BAT activity is regulated by sympathetic neural outflow from neural networks in the central nervous system (CNS). When norepinephrine (NE) is released from nerve terminals and binds beta-adrenergic receptors on brown adipocytes, an intracellular signaling cascade is initiated which leads to heat production through activation of the mitochondrial protein uncoupling protein 1 (UCP1). UCP1 functions to generate heat by dissipating chemical energy through a proton leak in the mitochondrial inner membrane resulting in adaptive (or non-shivering) thermogenesis^4,5^. In addition to classical BAT, beige or brite adipocytes found within white adipose depots appear in response to cold exposure and are capable of contributing to adaptive thermogenesis^6^.

Multiple peripheral signals converge upon the fundamental neural circuits controlling energy homeostasis and body temperature. Fibroblast growth factor 21 (FGF21) is a unique endocrine growth factor that regulates energy and nutrient homeostasis during various energetic and nutritional states^7,8^. FGF21 is a hormone that signals through a receptor complex consisting of a classical FGF receptor, FGFR1, and an obligate co-receptor, β-klotho^9,10^. Although signaling is activated via the FGF21:FGFR1 interaction, the initial binding of FGF21 to the β-klotho receptor is required for signaling activation^11^. Pharmacological administration of FGF21 increases energy expenditure and browning of adipose tissues *in vivo*^12–14^, and FGF21 treatment to human primary neck adipocytes increases both thermogenic gene expression and cellular respiration^15^. The physiological role of FGF21 in regulating energy expenditure, however, is less understood. FGF21 is produced and secreted by the liver in response to prolonged fasting, re-feeding, and macronutrient imbalance^7^. While an increase in circulating levels of FGF21 have been reported in mice exposed to cold^16^, other studies have not replicated this finding^17,18^. Mice lacking FGF21 fail to maintain core body temperature relative to control mice throughout a 3 day cold exposure and exhibit impairments in adaptive thermogenesis^17^. Recently, adipose-derived FGF21 was found to increase beiging of adipocytes through the recruitment of immune cells into subcutaneous white adipose depots thereby regulating body temperature^18^. In contrast to these studies, however, FGF21 was found to be dispensable for adaptation to prolonged cold exposure^19^. Here we show that FGF21 produced by the liver enters circulation upon the onset of cold and is acutely critical to maintain core body temperature. Furthermore, FGF21 signaling directly to adipose tissues is not required during cold exposure to maintain core body temperature. Instead, circulating FGF21 induced by cold acts centrally as a critical signal to fully activate sympathetic nerve activity to BAT in order to maintain body temperature. These data provide new physiological insight into the mechanisms regulating thermoregulation.

## Results

### Circulating FGF21 is increased during acute, but not chronic, cold exposure

**Figure 1:**
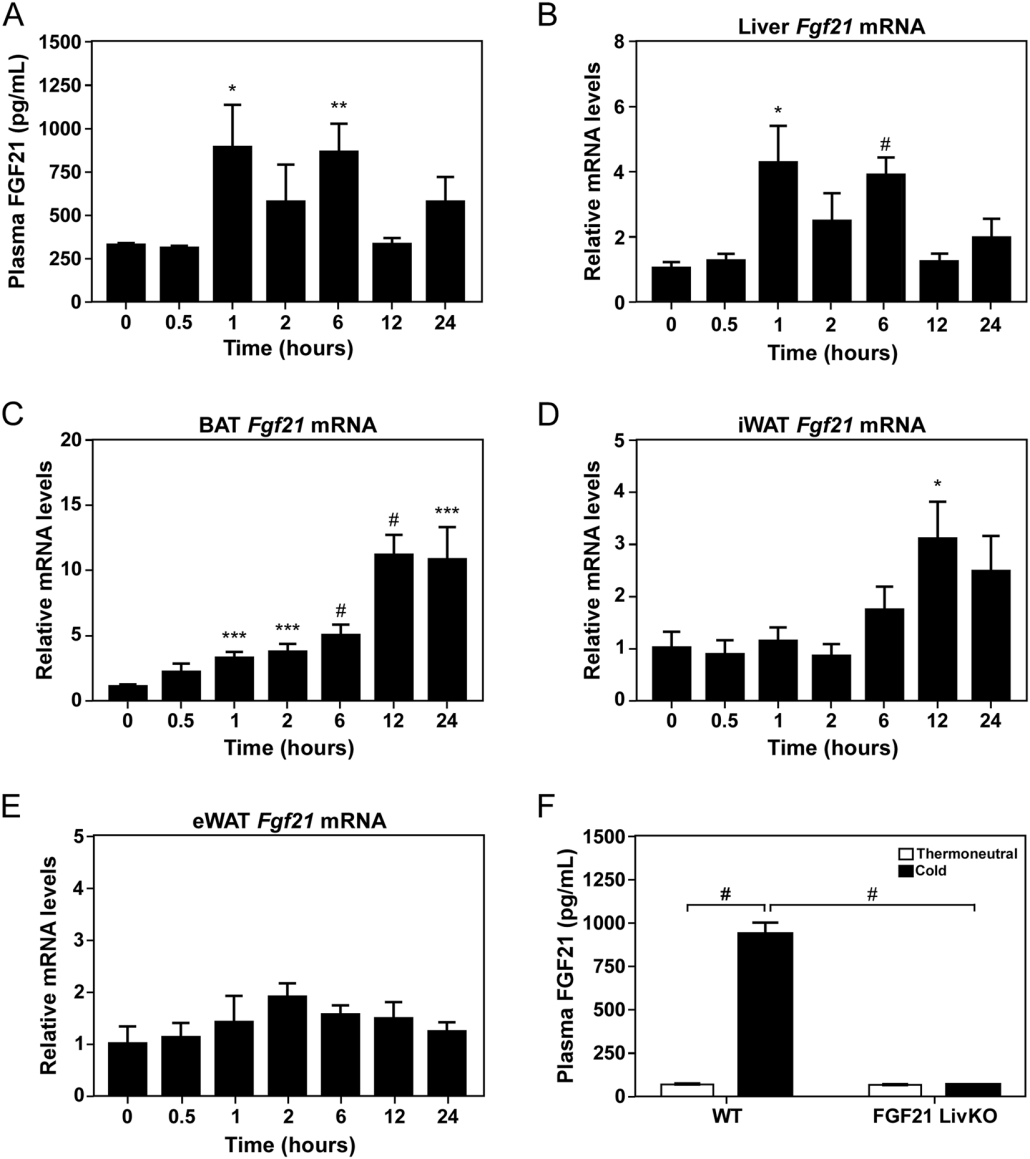
Acute cold exposure increases circulating levels of FGF21. (**A**) Plasma FGF21 levels in 12 week old C57Bl/6J male mice cold exposed for the indicated amount of time (n = 7/group). (B-E) *Fgf21* mRNA levels in (**B**) liver, (**C**) BAT, (**D**) iWAT and (**E**) eWAT from mice in (**A**). (**F**) Plasma FGF21 levels in 11–13 week old wild type (WT) and FGF21 LivKO male mice cold exposed for 1 hour (n = 5–6/group). Values are mean ± SEM; ^*^P ≤ 0.05; ^**^P ≤ 0.01; ^***^P ≤ 0.005; and ^#^*P* ≤ 0.001 compared to time 0 for (**A–E**) and relative to WT for (**F**).

To determine the effect of cold exposure on circulating FGF21 levels we performed a 24 hour time course by housing wild type mice at 4 °C. Plasma FGF21 levels were significantly increased in mice housed in cold for 1 and 6 hours (Fig. 1A) in conjunction with significant increases in hepatic *Fgf21* mRNA levels at these time points (Fig. 1B). BAT *Fgf21* mRNA was also significantly increased in mice housed in cold for 1 hour and progressively increased throughout the time course (Fig. 1C). In contrast, only modest changes were observed in *Fgf21* mRNA levels in iWAT and eWAT (Fig. 1D,E). To determine which tissue(s) contribute to circulating FGF21 levels, we measured plasma FGF21 levels from mice lacking FGF21 specifically in the liver (FGF21 LivKO). Consistent with the time course experiment, plasma FGF21 was significantly increased in wild type mice housed in cold for 1 hour and this induction of FGF21 was completely lost in FGF21 LivKO mice (Fig. 1F). These data demonstrate that circulating FGF21 levels derived from the liver are increased in response to acute cold exposure.

### Loss of hepatic FGF21 results in reduced core body temperature during cold exposure

**Figure 2:**
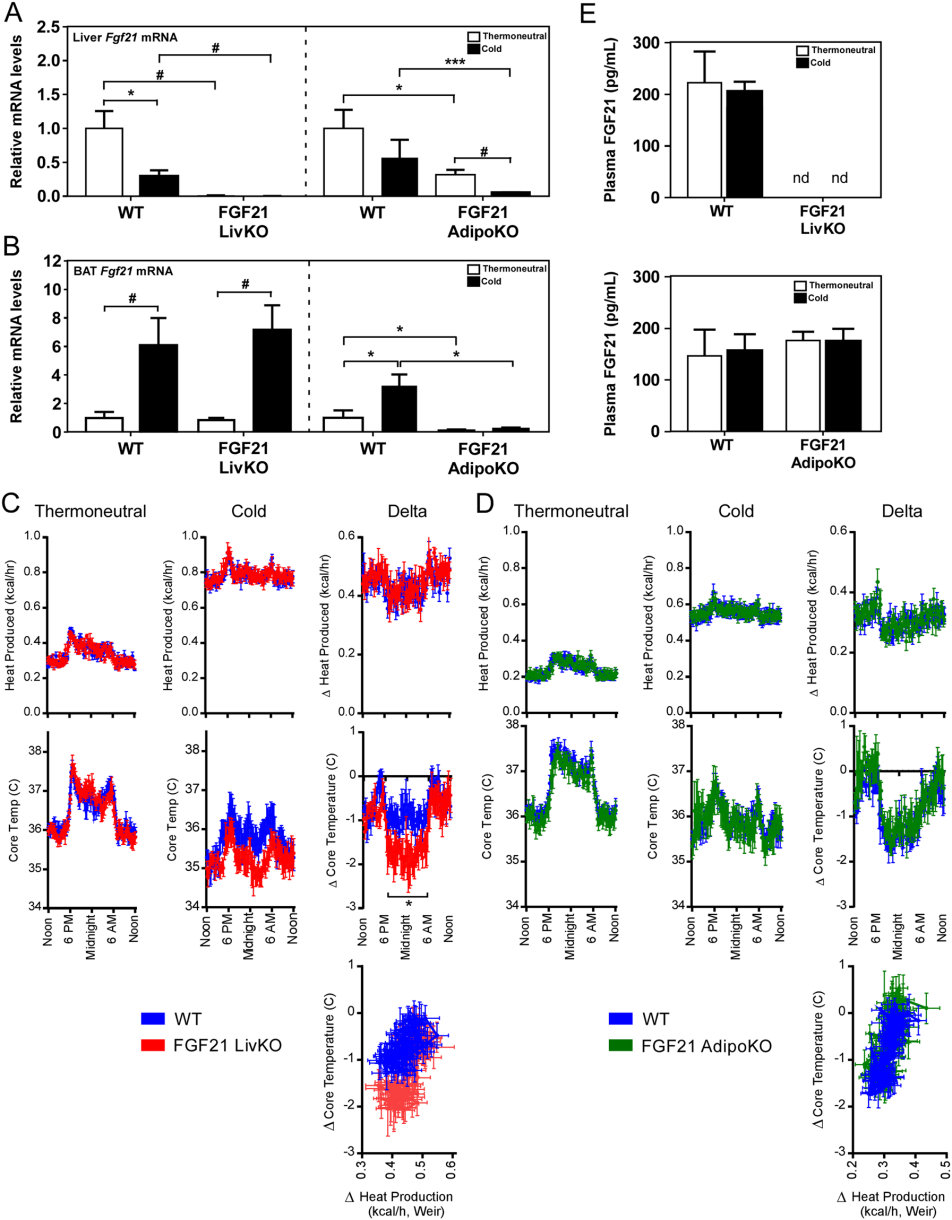
Hepatic FGF21 production is critical to maintain core body temperature during cold exposure. (**A**) Liver and (**B**) brown adipose tissue (BAT) *Fgf21* mRNA levels in 11–13 week old wild type (WT), FGF21 LivKO and FGF21 AdipoKO male mice housed at thermoneutrality or following 3 days of cold exposure. (**C**,**D**) Core body temperature (°C) and heat production (kcal/h) during thermoneutral and cold phases of 11–13 week old male FGF21 LivKO (**C**) and FGF21 AdipoKO (**D**) mice relative to WT controls (n = 6–8/group). Heat production by hour is estimated by the modified Weir equation. Core body temperature (°C) is also plotted against change in heat production (kcal/h). Change in core body temperature and heat production relative to time of day is also presented for FGF21 LivKO (**C**) and FGF21 AdipoKO (**D**) mice compared to WT controls (n = 6–8/group). For **C**, Genotype: *P* = 0.0866, Interaction between genotype and time of day: *P* = 0.0361 by 2-way RM ANOVA. (**E**) Plasma FGF21 levels from FGF21 LivKO and FGF21 AdipoKO mice shown in (**C**,**D**). Values are mean ± SEM; ^*^P ≤ 0.05; ^**^P ≤ 0.01; ^***^P ≤ 0.005; and ^#^*P* ≤ 0.001 compared to wild type mice; nd = not detected.

FGF21 total knockout (KO) mice housed at 4 °C for 3 days were previously shown to exhibit defects in adaptive thermogenesis in response to cold^17^. Based on our profiling data (Fig. 1), we hypothesized that loss of circulating FGF21 would result in impaired thermogenesis and an inability to maintain body temperature during cold exposure. To directly test this, we assessed changes in core body temperature via telemetry in mice specifically lacking FGF21 in liver (FGF21 LivKO) or adipose tissues (FGF21 AdipoKO). To validate our animal models, we measured hepatic and adipose *Fgf21* mRNA levels in FGF21 LivKO mice, FGF21 AdipoKO mice, and their respective littermate controls. As shown in Fig. 2, *Fgf21* mRNA expression is ablated specifically in the liver of FGF21 LivKO mice and specifically from adipose tissue of FGF21 AdipoKO mice (Fig. 2A,B). Following recovery from telemeter implantation, mice were housed at thermoneutrality for 6 days before the temperature was lowered to 4 °C for 3 days of cold exposure following a similar experimental paradigm used previously with FGF21 total knockout mice^17^. No significant differences in core body temperature were observed at thermoneutrality in FGF21 LivKO or FGF21 AdipoKO mice compared to wild type mice (Fig. 2C,D). However, in response to acute cold exposure, the change in core body temperature of FGF21 LivKO mice was significantly greater compared to wild type littermates, resulting in lower core body temperatures particularly during the dark cycle (Fig. 2C). In contrast, this effect on core body temperature was not observed between FGF21 AdipoKO and wild type littermates during cold exposure (Fig. 2D). Importantly, the reduction in core body temperature observed in FGF21 LivKO mice was not due to differences in body weight, food intake, or physical activity (Supplementary Fig. 1D–I). In addition, the greater drop in core body temperature of FGF21 LivKO mice was not due to changes in circulating energy substrate (glucose or lipid) availability (Supplementary Table 1) or substrate utilization (Supplementary Fig. 1A–C). Similar to the lack of induction of FGF21 after 24 hours, circulating FGF21 levels were not increased following 3 days of cold exposure (Fig. 2E), consistent with a previous report^17^. Finally, we measured *Ucp1* mRNA and other thermogenic gene expression in BAT of FGF21 LivKO and FGF21 AdipoKO mice compared to littermate controls. Thermogenic gene expression was similarly induced in FGF21 LivKO and FGF21 AdipoKO mice compared to littermate controls in response to cold (Supplemental Fig. 3A–H) and the ability to produce heat was similar between genotypes and their littermate controls (Fig. 2C,D). These data demonstrate that FGF21 produced by the liver is important to maintain core body temperature during acute cold exposure but is independent of changes in circulating metabolites or any alterations in BAT thermogenic gene expression.

### FGF21 signaling to adipose tissues is dispensable for maintaining core body temperature during acute cold exposure

**Figure 3:**
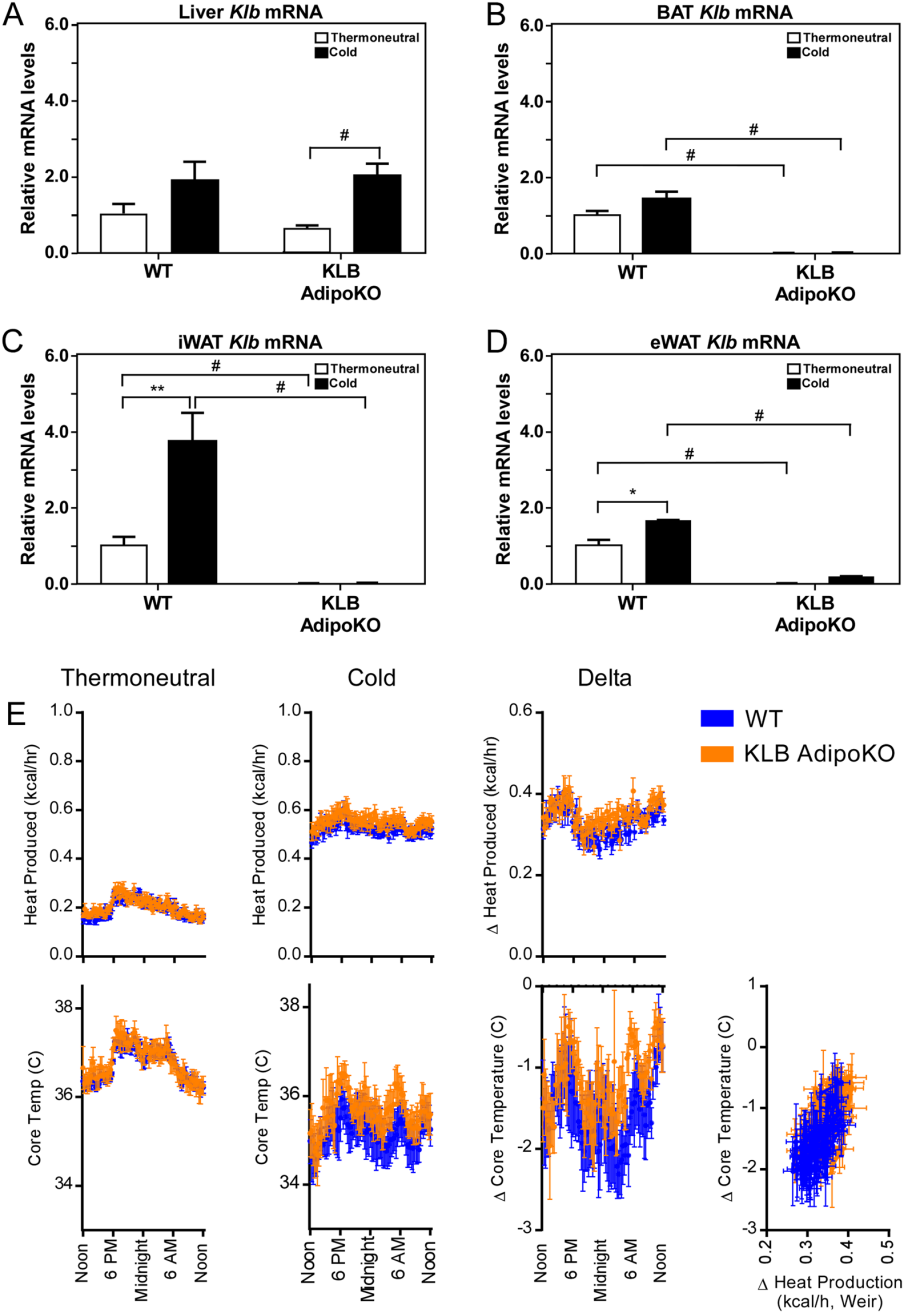
Loss of FGF21 signaling to adipose tissues does not affect core body temperature during cold exposure. (**A**) Liver, (**B**) BAT, (**C**) iWAT and (**D**) eWAT *Klb* mRNA levels in 11–13 week old male wild type (WT) and KLB AdipoKO mice housed at thermoneutrality or following 3 days of cold exposure (n = 6–7/group). (**E**) Core body temperature (°C) and heat production (kcal/h) during thermoneutral and cold phases of 11–13 week old male KLB AdipoKO mice relative to WT controls (n = 6–7/group). Heat production by hour is estimated by the modified Weir equation. Core body temperature (°C) is also plotted against change in heat production (kcal/h). Change in core body temperature and heat production relative to time of day is also presented for KLB AdipoKO mice compared to WT controls (n = 6–7/group). Values are mean ± SEM; ^*^P ≤ 0.05; ^**^P ≤ 0.01; ^***^P ≤ 0.005; and ^#^*P* ≤ 0.001 compared to wild type mice.

FGF21 signaling to adipose tissue is important for some of its metabolic function^20,21^, and adipocyte-derived FGF21 has been shown to regulate thermogenesis through autocrine/paracrine mechanisms^17,18^. Therefore, we next examined whether FGF21 signaling to adipose tissues in necessary for maintaining core body temperature during cold exposure by using mice that lack the obligate FGF21 co-receptor, β-klotho, specifically in adipose tissues (KLB AdipoKO) mice^20^. As shown in Fig. 3, β-k*lotho* mRNA expression is unchanged between the livers of wild type and KLB AdipoKO mice (Fig. 3A), but is abolished specifically in adipose tissues (Fig. 3B–D). KLB AdipoKO mice and wild type littermates were implanted with core telemeters and underwent the same cold exposure protocol described for FGF21 LivKO and AdipoKO mice (Fig. 2). Surprisingly, KLB AdipoKO mice had a slightly improved ability to defend core body temperature in response to cold compared to wild type littermates (Fig. 3E). No significant differences in body weight, food intake, or physical activity (Supplementary Fig. 4A–I) was observed between groups. However, KLB AdipoKO mice had a slight increase in heat production in response to cold (Fig. 3E). These data demonstrate that FGF21 signaling directly to adipose tissues is not necessary to maintain core body temperature during acute cold exposure.

### Hepatic FGF21 is important for maximal induction of BAT sympathetic nerve activity (SNA) during cold exposure

**Figure 4:**
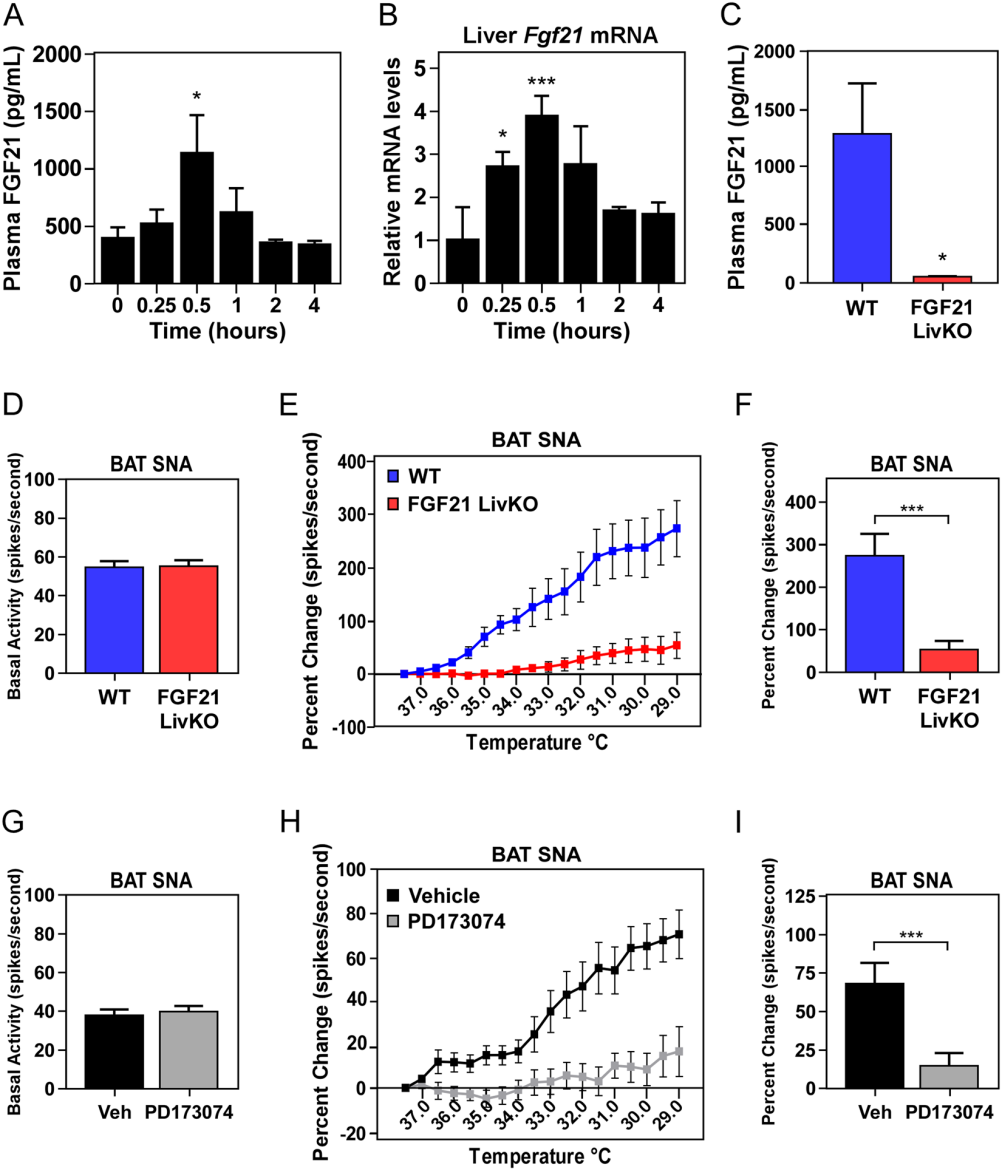
Liver-derived FGF21 regulates sympathetic nerve activity to BAT during cold exposure. (**A**) Plasma FGF21 levels and (**B**) hepatic *Fgf21* mRNA levels in 12–14 week old male wild type (WT) C57Bl/6J mice administered norepinephrine (1 mg/kg) for the indicated time (n = 8/group). (**C**) Plasma FGF21 levels in WT and FGF21 LivKO mice 30 min post-norepinephrine injection (n = 5–9/group). (**D**) Basal SNA to brown adipose tissue (BAT) in 12–14 week old male WT and FGF21 LivKO mice at ambient temperature and (**E**) during incremental cooling (n = 6–7/group). (**F**) Final percent change in BAT SNA in wild type and FGF21 LivKO mice at the final cooling point of 29 °C. (**G**) Basal SNA to BAT in 13 week old male WT mice administered ICV vehicle or PD173074 (25 µg) or (**H**) during incremental cooling (n = 10–11/group). (**I**) Final percent change in BAT SNA at 29 °C in wild type mice administered vehicle or PD173074. Values are mean ± SEM; ^*^P ≤ 0.05; ^**^P ≤ 0.01; and ^***^P ≤ 0.005 compared to time 0 for (**A**,**B**) and relative to wild type mice (**C–I**).

Cold exposure is a significant physiological stressor which activates a series of peripheral and central mechanisms required to prevent hypothermia^3^. Since circulating FGF21 levels are increased by acute cold (Fig. 1A) and FGF21 LivKO mice exhibit significantly lower core body temperatures following acute cold exposure (Fig. 2C), we tested the hypothesis that circulating FGF21 increases as part of an acute adaptative response to cold. Similar to the cold exposure time course, plasma FGF21 and hepatic *Fgf21* mRNA levels were also significantly increased in wild type mice acutely after peripheral administration of norepinephrine (Fig. 4A,B). This induction of plasma FGF21 levels, however, was lost when FGF21 was deleted specifically from the liver (FGF21 LivKO) (Fig. 4C), indicating that the liver produces circulating FGF21 in response to acute stress signals such as norepinephrine and acute cold.

To determine why FGF21 LivKO fail to properly maintain core body temperature, we examined whether FGF21 may regulate core body temperature by physiologically altering blood flow, heat loss, or sympathetic nerve activity (SNA). Importantly, loss of FGF21 from the liver did not affect mean arterial pressure or heart rate (Supplementary Fig. 5A,B), and administration of recombinant FGF21 did not affect tail temperature which is an important temperature regulatory mechanism in rodents^22^ (Supplementary Fig. 5C,D). Instead, thermal imaging in conscious, unrestrained, wild type mice revealed that FGF21 treatment caused a significant increase in body surface temperature localized to the interscapular brown adipose tissue (Supplementary Fig. 5D,E), suggesting increases in brown adipose thermogenesis. Previous studies using pharmacological administration of FGF21 demonstrated that FGF21 is sufficient to increase SNA to adipose tissues^23,24^. To test whether liver-derived FGF21 physiologically regulates BAT SNA, we directly measured SNA from sympathetic nerves subserving BAT in FGF21 LivKO mice at ambient temperature and in response to acute cold. No differences in basal BAT SNA were detected between FGF21 LivKO mice and wild type littermates (Fig. 4D). However, while SNA to BAT of WT mice markedly increased as expected in response to acute, incremental cooling, this effect was significantly attenuated in FGF21 LivKO mice (Fig. 4E,F). These data indicate that physiological induction of liver-derived FGF21 is important for the full stimulation of SNA to BAT during acute cold exposure.

To examine whether circulating FGF21 acts centrally to activate SNA to BAT during cold exposure, we administered either vehicle (DMSO) or the FGF receptor inhibitor PD173074 directly into the brain of WT mice via an intracerebroventricular (ICV) cannula. BAT SNA was then recorded during cooling. PD173074 had no effect on basal BAT SNA (Fig. 4G) but prevented the increase in BAT SNA evoked by cold (Fig. 4H,I). Collectively, our data demonstrate that in response to cold hepatic production of FGF21 is acutely increased to signal to the CNS the need to increase BAT SNA as part of a physiological response to defend core body temperature.

## Discussion

Although the pharmacological effects of FGF21 on energy expenditure have been well documented^8^, few studies have explored the physiological functions of endogenous FGF21 during cold. In this study, we show that circulating FGF21 is acutely produced from the liver in response to a cold challenge. Furthermore, the loss of circulating FGF21 impairs the ability to maintain core body temperature in response to cold exposure. These phenotypic data are consistent with those of Fisher *et al*., which observed reduced core body temperature in FGF21 total KO mice during cold^17^. Based on limited tissue and plasma FGF21 analyses^17^, Fisher *et al*. concluded that FGF21 was derived from adipose tissue to regulate adaptive thermogenesis. However, in this current study we utilized genetic FGF21 mouse models to determine that liver-derived, but not adipose-derived, FGF21 acutely regulates core body temperature in response to cold. Interestingly, while multiple studies have reported changes in *Fgf21* mRNA in adipose tissues of mice, it is questionable whether *FGF21* mRNA is expressed in human adipose tissue^25^. Although our studies involved a relatively short term cold exposure, a recent study utilizing FGF21 total KO mice concluded that FGF21 is not required for the defense of body temperature during prolonged cold exposure^19^. These findings, combined with the lack of induction of circulating levels of FGF21 that we observed in response to extended cold (Fig. 2D) and as reported by Fisher *et al*.^17^, support the conclusion that FGF21 is not involved in the physiological regulation of defense of body temperature during long-term cold exposure.

Multiple lines of evidence suggest that FGF21 signals directly to adipose tissue to regulate adaptive thermogenesis^8^, including the ability of FGF21 to increase thermogenic gene expression in primary adipocytes *in vitro*^15,17^. In contrast to these data, recent *in vivo* studies have demonstrated that CNS action of FGF21 regulates systemic energy homeostasis. For example, loss of β-klotho from the CNS^24^, but not adipose tissues^20,21^, abolishes FGF21-mediated increases in energy expenditure and weight loss. In addition, central administration of FGF21 is sufficient to increase SNA to adipose tissues^23,24^ suggesting a central mechanism may underlie the physiological effects of FGF21 during acute cold exposure. In support of this, our data demonstrate that liver-derived FGF21 is induced acutely in response to cold and signals to the CNS to regulate SNA to BAT. In contrast, Huang *et al*. reported that adipose-derived FGF21 regulates adaptive thermogenesis through a CCL11-mediated recruitment of eosinophils to subcutaneous adipose tissue^18^. The explanation for the differences between studies may be due to differences in experimental design. While the Huang *et al*. study used rectal thermometers to assess body temperature at specific times^18^, our study utilized telemeters to continuously measure core body temperature independent of animal handling. Additionally, to delete FGF21 from adipose tissue our study utilized Adiponectin-Cre transgenic mice which expresses Cre recombinase specifically in adipocytes^26^, while the Huang *et al*. study used aP2-Cre transgenic mice^18^ which express Cre recombinase in adipose and non-adipose tissues^26,27^. We found that FGF21 is induced in the liver at an earlier time point during cold exposure than measured by Huang *et al*. In our studies, circulating FGF21 was significantly elevated one hour after the environmental chamber began cooling corresponding to the mice being housed at 4 °C for approximately 20 min. Thus, the induction in plasma FGF21 levels occurred prior to the earliest time point examined in the studies of Huang *et al*. Finally, in contrast to our data, a study by Hill *et al*. found that loss of FGF21 had no effect on body temperature during 6 hours of cold exposure^28^. However, this study occurred under conditions of food restriction^28^, and it is unclear whether that may contribute to the observed differences between studies.

Our data indicate that loss of FGF21 derived from the liver, but not adipose tissues, disrupts the normal relationship between heat production, dissipation, and retention. Experiments to analyze the metabolic activity of our different animal models were performed by simultaneously measuring aerobic heat production and core body temperature. The results from these two independent measurements, however, revealed a divergence in endpoints with FGF21 LivKO mice exhibiting a decrease in core body temperature despite normal heat production. Thermal imaging studies revealed that FGF21 does not promote heat dissipation (Supplementary Fig. S5E), although many other sources of heat dissipation could not be accounted for in our experiments during thermoneutral and cold conditions including evaporative water loss (exhaled water vapor, saliva spreading), dry heat loss in expired air, direct heat conducted through contact with the floor of the cage, and energy lost to the urine and feces, etc. Additionally, changes in anaerobic metabolism, undetectable via indirect calorimetry, could change core body temperature. Nevertheless, we observed a clear divergence between aerobic heat production versus core body temperature during cold exposure in FGF21 LivKO mice relative to control mice. To reconcile this, we postulate that core body temperature in FGF21 LivKO mice is reduced despite “normal” levels of heat production because mice lacking circulating FGF21 fail to properly perceive that the heat production achieved is insufficient to maintain their core body temperature. In the FGF21 LivKO mice, a “normal” rate of heat production is deemed normal based on the rate of heat production of control mice. However, for the FGF21 LivKO mice, this heat production is not normal because it inappropriately responds to the true biological stimulus of core body temperature, which is inappropriately low in these mice. These results, in concert with the findings that (i) BAT SNA responses to cooling are altered in these animals and (ii) tail skin vasodilatory responses appear to be largely normal in wild type animals in response to FGF21, suggests that FGF21 from the liver seems to modulate afferent input or integration of thermoregulatory stimuli, as opposed to modulating efferent heat production or dissipation-controlling autonomic activity directly.

An additional divergent result from our studies is that FGF21 LivKO mice fail to acutely increase BAT SNS outflow in response to cold despite cold-induced increases in BAT UCP1 and heat production. We can only speculate explanations for this observation, but possibilities include life-long adaptation to reduced autonomic tone or major differences in thermogenic capacity (e.g., mitochondrial capacity, etc.) of FGF21 LivKO mice. Additional studies are necessary to test these possibilities. Interestingly, loss of hepatic, and thus circulating, FGF21 levels resulted in reduced core body temperatures primarily during the dark cycle. When induced, hepatic and plasma FGF21 levels exhibit a circadian expression profile with the highest levels occurring at the initiation of the dark cycle^29,30^. This diurnal rhythm of FGF21 also occurs in humans in response to mild cold exposure^31^. Thus, the observed effects on core body temperature occur when FGF21 might be predicted to be elevated. Finally, while we observed differences in SNA to BAT, we cannot exclude the possibility that changes in shivering thermogenesis, which is rapidly activated in mammals upon the onset of cold, is not altered by loss of FGF21 since it was not evaluated in our studies. Future studies are needed to fully delineate the precise central pathways mediating these effects of FGF21 on core body temperature. Together, our data support a physiological role for hepatic production of FGF21 in response to acute cold to enhance SNA to BAT to maintain core body temperature.

## Material and Methods

### Animals

FGF21^fl/fl^ ^32^, FGF21 liver-specific knockout (FGF21 LivKO)^33^, FGF21 adipose-specific knockout (FGF21 AdipoKO)^33^, KLB^fl/fl^^34^, and β-klotho adipose-specific knockout mice (KLB AdipoKO)^20^ have been described previously. All mice used in these studies were male littermates on a C57Bl/6J genetic background maintained on standard chow (2920X; Envigo). For experiments, all mice were housed individually on a 12:12 hour light:dark cycle with ad libitum access to chow and water at 22 °C unless otherwise noted. For cold experiments, mice were acclimated to wire cage bottoms for at least 5 days prior to cold exposure and were maintained on wire bottoms throughout cold exposure experiments. All mouse experiments presented in this study were conducted in accordance with the animal research guidelines from NIH and were approved by the University of Iowa IACUC.

### Cold exposure time course

Twelve-week old, C57Bl/6J male mice were individually housed in a rodent environmental chamber (Power Scientific) at 30 °C for 72 hours. At time 0 the housing temperature was set to 4 °C to begin the time course. Thus, time 0 is the time at which the environmental chamber started cooling. The chamber reached 4 °C after 40 minutes. At each time point mice were sacrificed by decapitation for the collection of trunk blood and tissues.

### Assessment of core body temperature

Core body temperature telemeters (Respironics, G2 E-Mitter) were surgically implanted into the abdominal cavity and mice were then allowed to recover for 5 days post-surgery. Mice were then individually housed in an OxyMax Comprehensive Lab Animal Monitoring System (CLAMS, Columbus Instruments International) at 28 °C for 6 days followed by 4 °C for 3 days, similar to a previous study^17^. Core body temperature was recorded every 17 minutes throughout the experiment, along with heat production and respiratory exchange ratio (RER) as estimated by respirometry, food intake, and physical activity as estimated by photoelectric beam breaks in the X + Y plane. To compare plasma parameters between cold exposed mice and mice housed at thermoneutrality, separate cohorts of wild type, FGF21 LivKO and FGF21 AdipoKO mice were housed at thermoneutrality.

### *In Vivo* Norepinephrine Studies

Male C57Bl/6J mice 12–14 weeks of age were individually housed at 30 °C for 3 days before receiving an intraperitoneal (i.p.) injection of norepinephrine (1 mg/kg) or saline vehicle. Mice were euthanized while at 30 °C at the indicated time points and blood and tissues collected for analysis.

### Continuous sympathetic nerve activity, heart rate, and blood pressure recording

To test the effect of acute cold on SNA, mice were anesthetized and prepared for nerve recording as previously described^35^. Body temperatures were measured via rectal thermometer. Blood pressure and heart rate were measured continuously throughout the experiment as described^36^. A 30 minute baseline SNA was obtained at core body temperature of 37.5 °C. Mice were then cooled by lowering the surgical platform temperature and SNA was recorded continuously during the drop in body temperature. For the FGFR inhibitor experiments, cannulas were surgically implanted into the lateral ventricle of 12-week old male C57Bl/6J mice at least one week before the SNA studies. After baseline SNA recording, mice received an ICV injection of either vehicle (DMSO) or PD173074 (25 ug; Tocris) and were then exposed to cold while SNA was recorded as described above.

### Gene expression

All tissues were flash frozen in liquid nitrogen and stored at −80 °C prior to analysis. RNA was isolated using Trizol reagent per manufacturer’s instructions. Single stranded cDNA was synthesized using 2 µg RNA (AB High Capacity cDNA kit) and qPCR performed using SybrGreen (Invitrogen).

### Plasma analyses

Blood was collected in 300K2E microvettes (Sarstedt) and spun at 3000 rpm for 30 minutes at 4 °C to separate plasma. Mouse plasma FGF21 levels were measured using a commercially available ELISA (Biovendor). Plasma glucose and NEFAs were determined using colorimetric assays (Wako). Plasma triglycerides and cholesterol were also measured using colorimetric assays (Infinity^TM^, Thermo Scientific). All measurements were performed according to the manufacturer’s instructions.

### Infrared imaging

Wild-type male C57Bl/6J littermate mice 14 weeks of age were injected i.p. once daily with vehicle or recombinant human FGF21 (1 mg/kg BW). Immediately following injection, tail temperature was assessed using a dual channel Traceable Expanded Range Probe (Fisher Scientific). Approximately 4 hours after injections, body surface temperature was imaged in fully awake, unrestrained mice using a high-resolution infrared camera (A655sc Thermal Imager; FLIR Systems, Inc.) as described^37^.

### Statistical analysis

Data were analyzed using Microsoft Excel and GraphPad Prism 7. All data are presented as mean ± SEM, and p < 0.05 was considered statistically significant. Differences between groups or relative to time 0 were determined via Student’s t-test or ANOVA where indicated.

## Supplementary information

Supplementary Information

## Data Availability

The datasets generated during and/or analyzed during the current study are available from the corresponding authors on reasonable request.
