# Supplementary material for: Liver Derived FGF21 Maintains Core Body Temperature During Acute Cold Exposure
Source: Sci Rep. 2019 Jan 24;9:630. doi: 10.1038/s41598-018-37198-y (PMC6345819; doi:10.1038/s41598-018-37198-y)
Supplement: Supplementary file 1 — Supplementary Information [file 41598_2018_37198_MOESM1_ESM.pdf]

Scientific Reports
Supplementary Information

# Liver Derived FGF21 Maintains Core Body Temperature During Acute Cold Exposure

Magdalene Ameka, Kathleen R. Markan, Donald A. Morgan, Lucas D. BonDurant, Sharon O. Idiga, Meghan C. Naber, Zhiyong Zhu, Leonid V. Zingman, Justin L. Grobe, Kamal Rahmouni, and Matthew J. Potthoff

**Supplementary Table 1: Plasma parameters of WT, FGF21 LivKO and FGF21 AdipoKO mice at thermoneutrality and cold exposed.** Data are shown as mean ± S.E.M and were analyzed via t-test against respective WT control group (n = 4-12/group).

| | **Thermoneutrality** | | | | **Cold Exposed** | | | |
|---|---|---|---|---|---|---|---|---|
| **Plasma Parameter** | **WT** | **FGF21 LivKO** | **WT** | **FGF21 AdipoKO** | **WT** | **FGF21 LivKO** | **WT** | **FGF21 AdipoKO** |
| **Glucose (mg/dL)** | 161.40 ± 18.43 | 158.06 ± 5.84 | 141.41 ± 14.47 | 161.92 ± 7.33 | 175.05 ± 12.61 | 198.36 ± 20.47 | 170.64 ± 5.54 | 176.03 ± 11.67 |
| **NEFAs (mmol/L)** | 1.65 ± 0.16 | 1.64 ± 0.10 | 0.61 ± 0.13 | 0.57 ± 0.08 | 1.28 ± 0.33 | 1.01 ± 0.22 | 1.66 ± 0.43 | 1.19 ± 0.21 |
| **Triglycerides (mg/dL)** | 141.09 ± 20.56 | 173.30 ± 17.12 | 117.09 ± 22.17 | 139.71 ± 12.65 | 40.30 ± 2.25 | 38.27 ± 3.03 | 34.22 ± 1.40 | 40.67 ± 4.63 |
| **Cholesterol (mg/L)** | 113.93 ± 10.26 | 128.05 ± 4.52 | 109.66 ± 15.16 | 128.92 ± 5.90 | 103.45 ± 2.65 | 107.36 ± 2.68 | 81.02 ± 3.96 | 86.98 ± 5.41 |

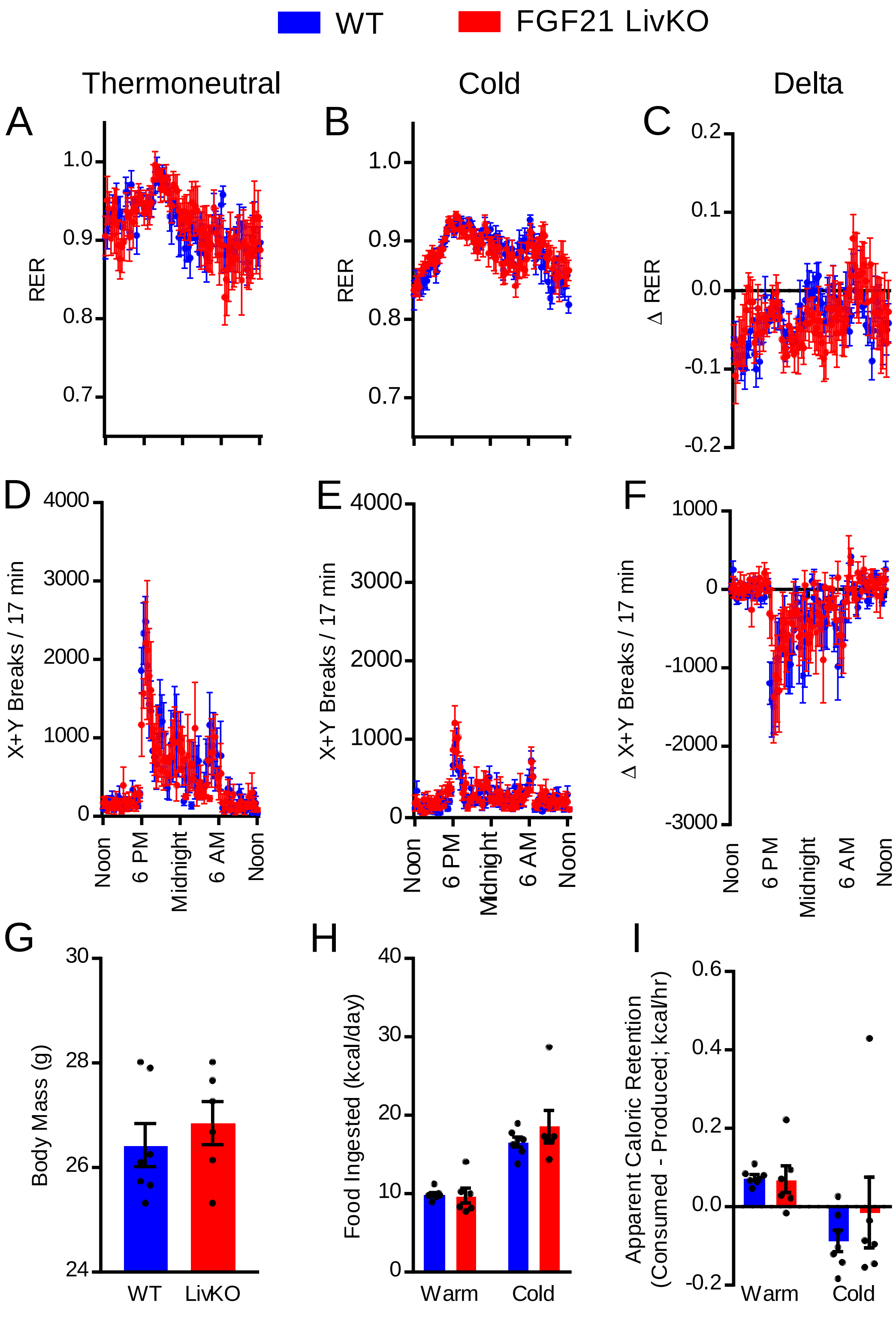


**Supplementary Figure 1**

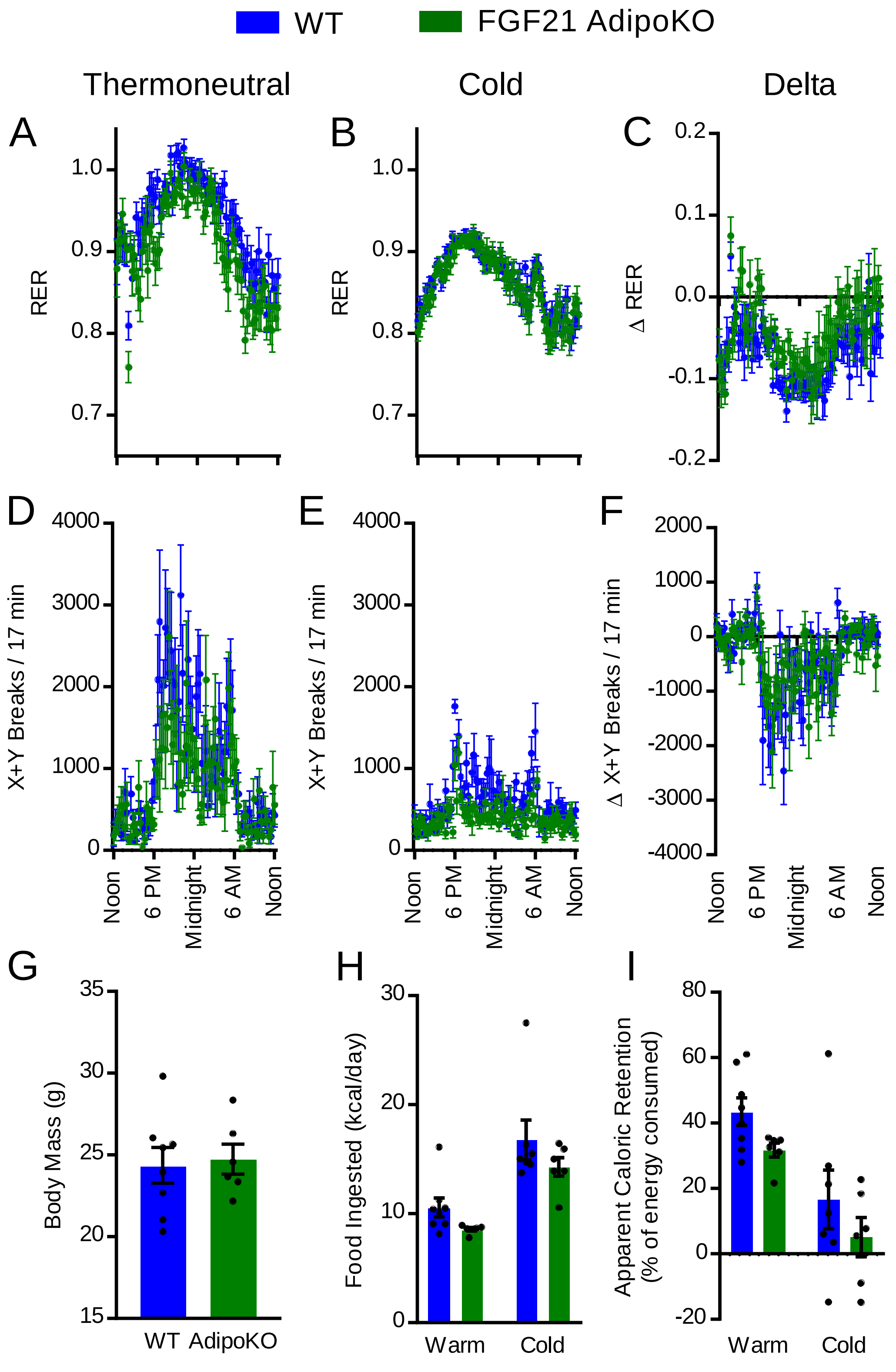


**Supplementary Figure 2**

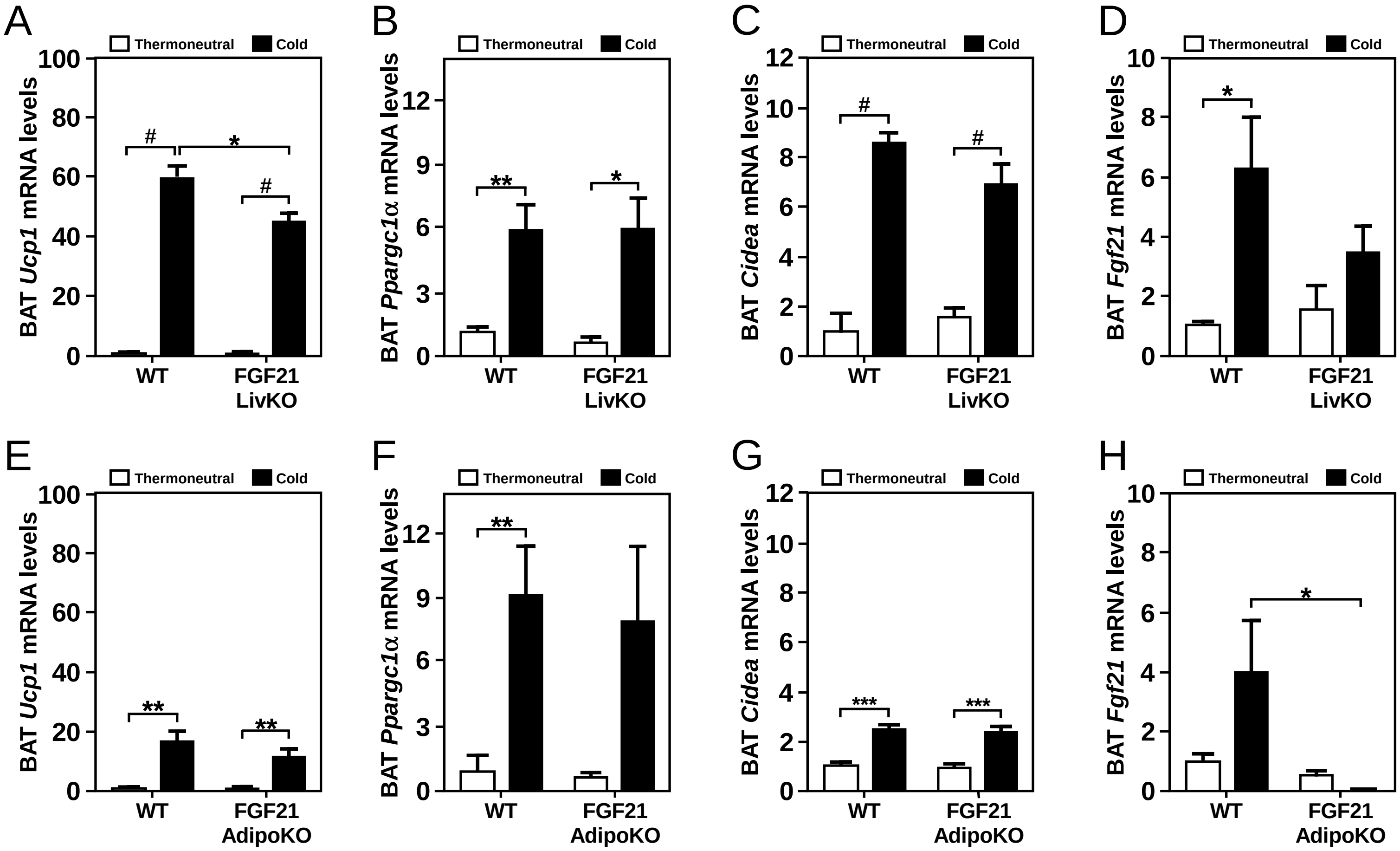


**Supplementary Figure 3**

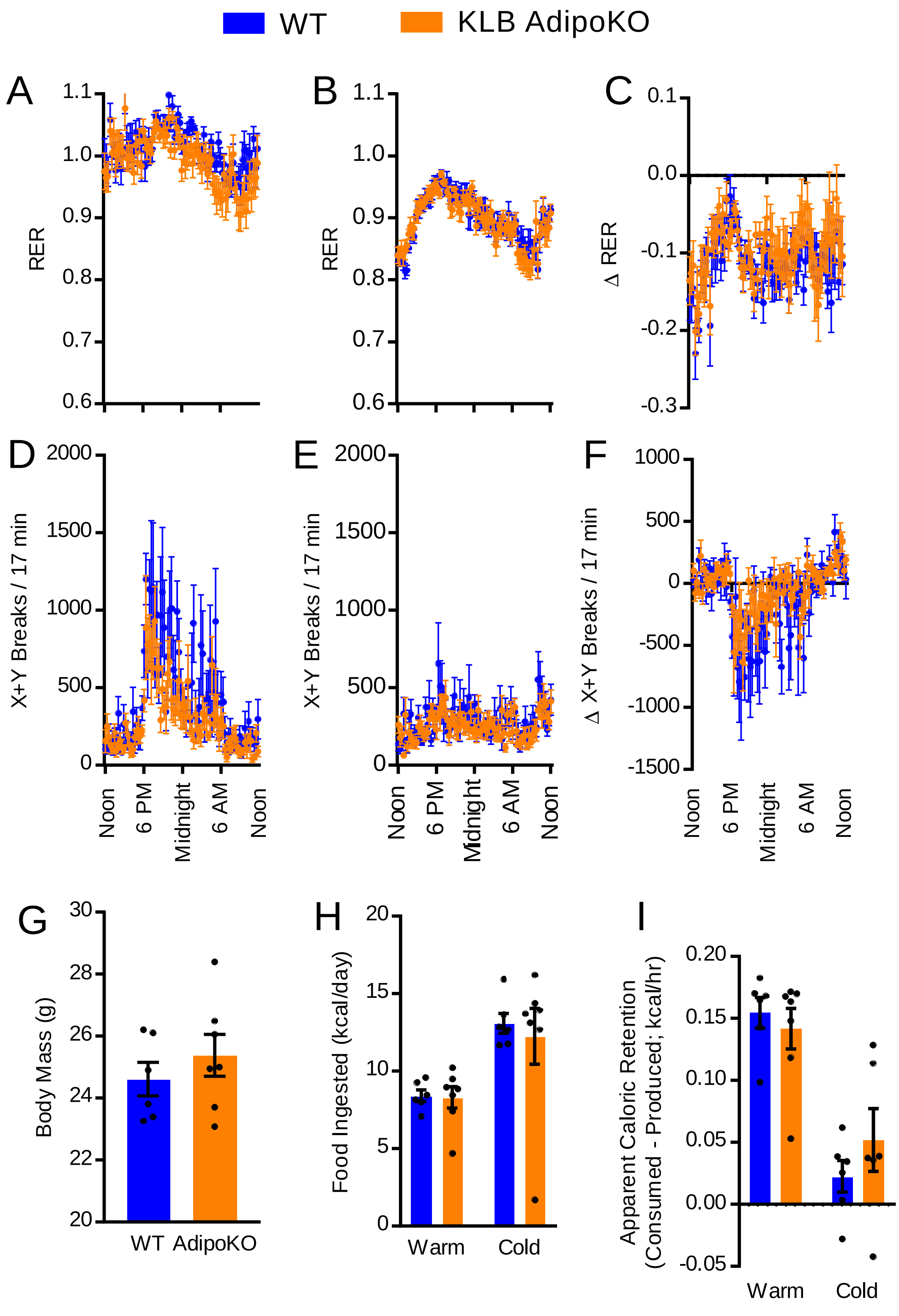


**Supplementary Figure 4**

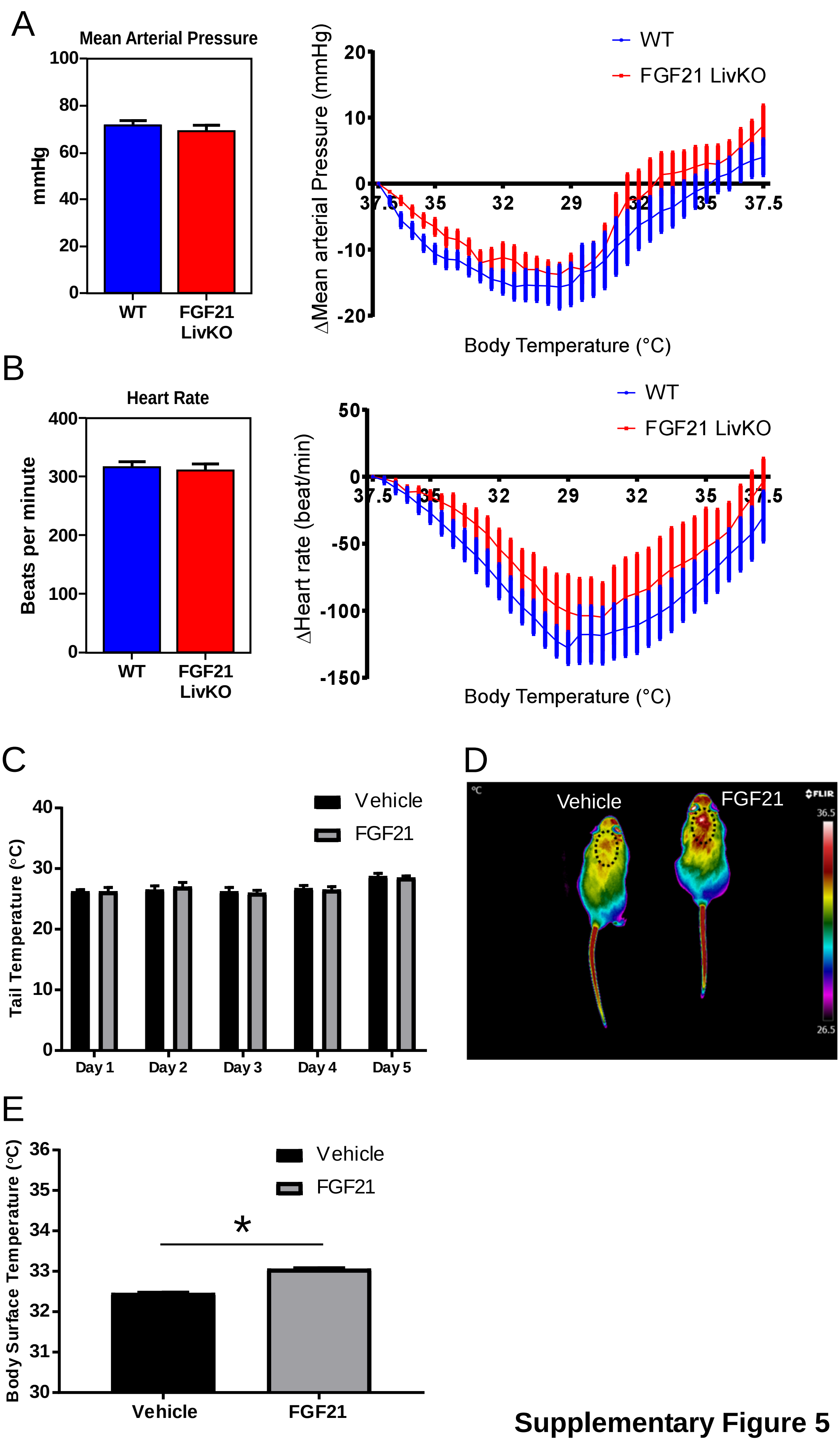


Supplementary Figure 5

## SUPPLEMENTARY FIGURE LEGENDS

**Supplementary Figure 1:** (A-I) Energy homeostasis during thermoneutral (28C) and cold (4C) exposures in FGF21 liver-specific knockout (FGF21 LivKO) and littermate control mice, analyzed using OxyMax (Columbus Instruments International). (A-C) Respiratory exchange ratio. (D-F) Total photoelectric beam interruptions in the X+Y plane. (G) Body masses. (H) Food consumed. (I) Apparent caloric retention, calculated as [calories ingested] minus [heat production]. For all panels, n = 7 control vs n = 6 FGF21 LivKO. Values are mean ± SEM.

**Supplementary Figure 2:** (A-I) Energy homeostasis during thermoneutral (28C) and cold (4C) exposures in FGF21 adipose-specific knockout (FGF21 AdipoKO) and littermate control mice, analyzed using OxyMax (Columbus Instruments International). (A-C) Respiratory exchange ratio. (D-F) Total photoelectric beam interruptions in the X+Y plane. (G) Body masses. (H) Food consumed. (I) Apparent caloric retention, calculated as [calories ingested] minus [heat production]. For all panels, n = 8 control vs n = 6 FGF21 AdipoKO. Values are mean ± SEM.

**Supplementary Figure 3:** Brown adipose tissue (BAT) thermogenic gene expression in (A-D) FGF21 LivKO mice and (E-H) FGF21 AdipoKO mice housed at thermoneutrality or cold exposed for 3 days (n = 6-7/group). Values are mean ± SEM; *, $P \leq 0.05$; ** $P \leq 0.01$; *** $P \leq 0.005$; and # $P \leq 0.001$ compared to wild type mice.

**Supplementary Figure 4:** (A-I) Energy homeostasis during thermoneutral (28C) and cold (4C) exposures in KLB adipose-specific knockout (KLB AdipoKO) and littermate control mice, analyzed using OxyMax (Columbus Instruments International). (A-C) Respiratory exchange ratio. (D-F) Total photoelectric beam interruptions in the X+Y plane. (G) Body masses. (H) Food

consumed. (I) Apparent caloric retention, calculated as [calories ingested] minus [heat production]. For all panels, n = 6 control vs n = 7 KLB AdipoKO. Values are mean ± SEM.

**Supplementary Figure 5:** Basal and change in mean arterial pressure (A), and basal and change in heart rate (B) of WT (FGF21$^{fl/fl}$) and FGF21 LivKO prior to and during incremental cooling for the recording of body temperature (n = 6-7/group). (C) Tail temperature in conscious, unrestrained wild type mice administered vehicle or FGF21 (1 mg/kg) via i.p. injection for the indicated time. (D) Representative image of thermal imaging of mice in (C). (E) Quantification of body surface temperature of mice in (D). Values are mean ± SEM; *, $P \leq 0.05$.
